# Tale of Two Cities: narrative review of oxygen

**DOI:** 10.12688/f1000research.130592.2

**Published:** 2023-05-09

**Authors:** Pranathi Gullapalli, Nicoletta Fossati, Dusica Stamenkovic, Muhammad Haque, Davide Cattano

**Affiliations:** 1 Department of Anesthesiology, McGovern Medical School UTHealth, Hosuton, USA; 2 Department of Anaesthesia, St George’s Hospital and Medical School, London, UK; 3 Military Medical Academy, University of Defense, Belgrade, Serbia; 4 Department of Neurology, McGovern Medical School UTHealth, Houston, USA

**Keywords:** oxygen therapy, cerebral autoregulation, cerebral ischemia, stroke, cerebral blood flow

## Abstract

The human brain contributes 2% of the body weight yet receives 15% of cardiac output and demands a constant supply of oxygen (O _2_) and nutrients to meet its metabolic needs. Cerebral autoregulation is responsible for maintaining a constant cerebral blood flow that provides the supply of oxygen and maintains the energy storage capacity. We selected oxygen administration-related studies published between 1975–2021 that included meta-analysis, original research, commentaries, editorial, and review articles. In the present narrative review, several important aspects of the oxygen effects on brain tissues and cerebral autoregulation are discussed, as well the role of exogenous O _2_ administration in patients with chronic ischemic cerebrovascular disease: We aimed to revisit the utility of O _2_ administration in pathophysiological situations whether or not being advantageous. Indeed, a compelling clinical and experimental body of evidence questions the utility of routine oxygen administration in acute and post-recovery brain ischemia, as evident by studies in neurophysiology imaging. While O _2_ is still part of common clinical practice, it remains unclear whether its routine use is safe.

## Introduction

The human brain accounts for only 2% of total body weight, yet receives about 15% of the entire cardiac output: with minimal capacity to store energy. It demands a constant supply of oxygen (O _2_) and nutrients to meet metabolic needs and maintain cerebral function. Even a short interruption in cerebral blood flow (CBF) can initiate a cascade of pathological events. ^1^^,^^2^

In order to maintain this equilibrium, cerebral autoregulation (CA) and hyperemia are responsible for regulating CBF: while hyperemia maintains localized microscopic blood flow by regulating global CA via constriction and dilation and maintaining CBF between 40 and 60 mL/100 g/min over a wide range of mean arterial pressure (MAP; 50–150 mmHg). ^3^^,^^4^ Outside this range and in the absence of other pathophysiological occurrences, hypotension can technically cause cerebral ischemia, whereas hypertension could lead to a hemorrhagic stroke: both can ultimately initiate a sequence of neuropathological events such as neuroinflammation, synaptic dysfunction, and neuronal death (excitotoxicity, apoptosis, necrosis, *etc.*, also known as programmed or not-programmed cell-death mechanisms). ^5^ In the present narrative review we will summarize some of the general physiological and pathophysiological mechanisms underscoring the role of CA in regulating CBF in various scenarios.

**Figure 1.**
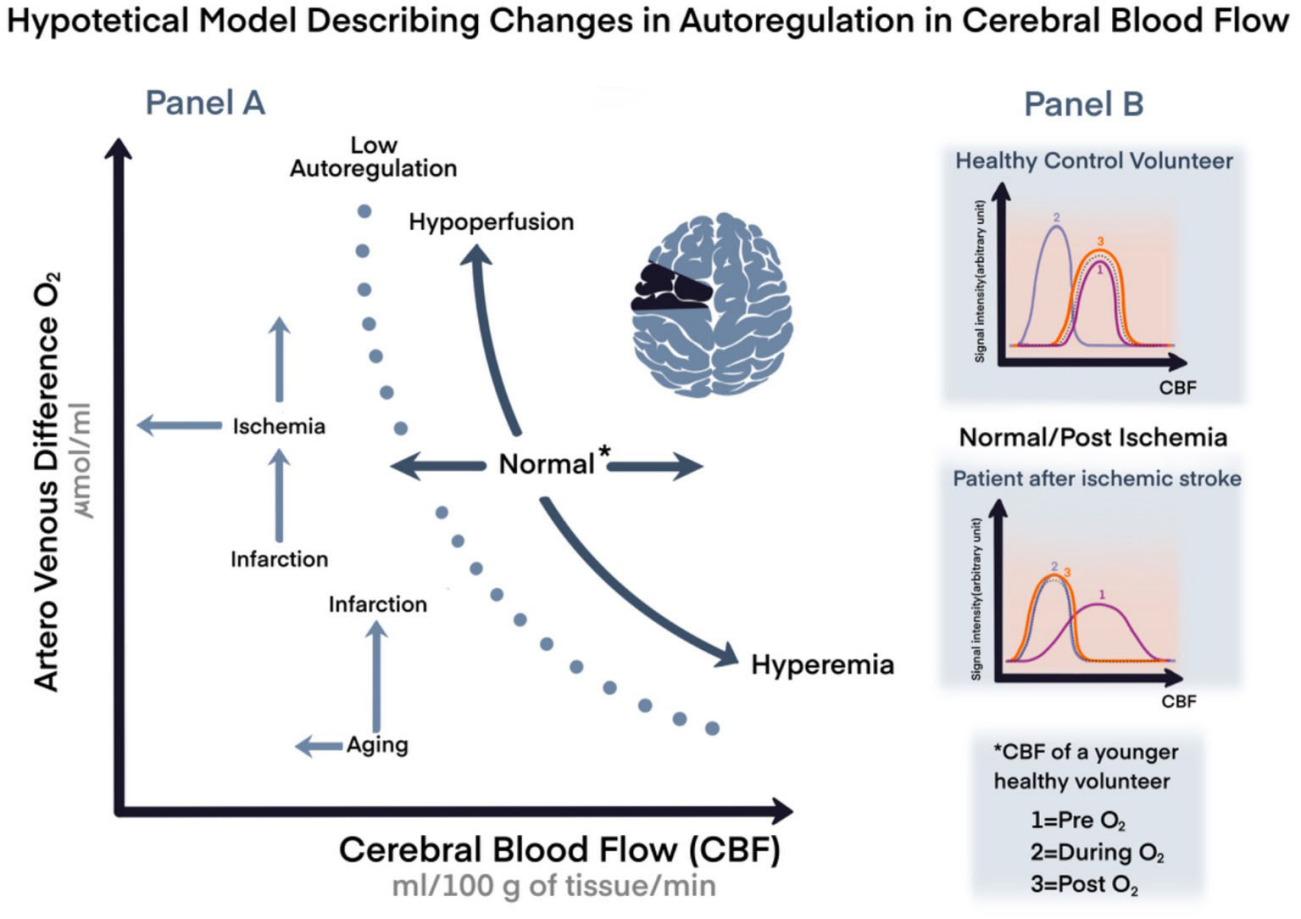
Hypothetical model representing changes in cerebral autoregulation during aging or ischemic events. Panel A. A representation of cardiovascular coupling during normal or stressful events with variation in age, and compensation mechanisms. Panel B. Hypothetical model comparing changes in cerebral blood flow (CBF) in healthy volunteer compared to a patient with chronic ischemic stroke by magnetic resonance imaging. *Arbitrary unit=signal intensity; CBF of a young volunteer; CBF: cerebral blood flow; O _2_: oxygen; 1: pre O _2_; 2: during O _2_; 1: post O _2_.

In a healthy brain, CA—alone and by far—prevents the development of either ischemia or hyperemia caused by MAP changes; however, CA capabilities decline with aging and/or may become impaired, for instance after cerebrovascular accidents. The brain, during these physiological and clinical scenarios is more vulnerable, and it is even further exposed to more damage than it would have been if exposed to the same situations when younger or with better reserves, we could say (hypothetical model in Figure 1).

**Figure 2.**
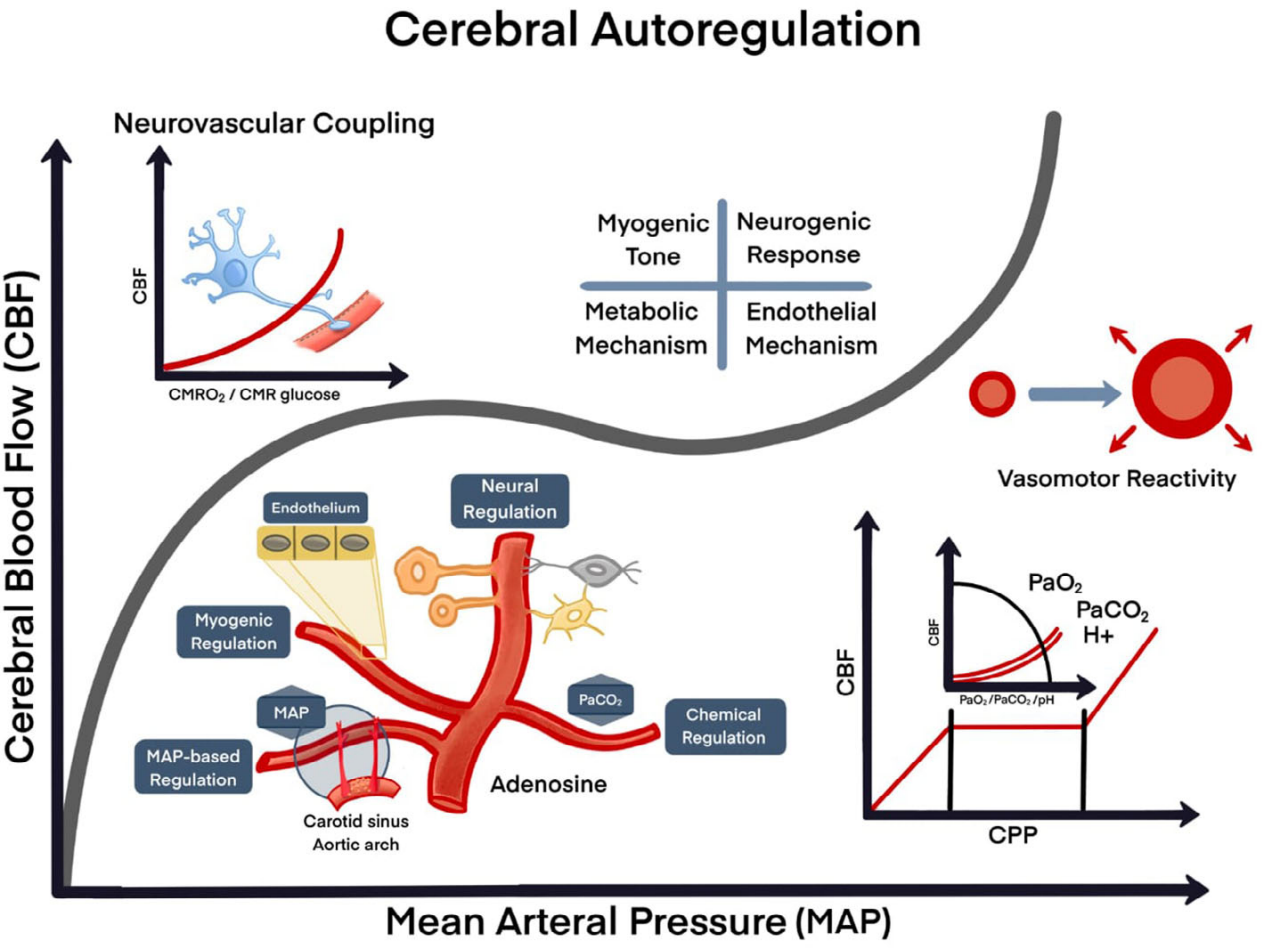
Model of cerebral autoregulation. A. Cellular interaction (neurons, endothelial cells and glial cells); B. Nutrients/energy (glucose/oxygen); C. Activity/changes of demand, physiological; D. Pathological (seizures, stroke, trauma). Four mechanisms control mechanisms: myogenic, neurogenic, metabolic and endothelial. CBF: cerebral blood flow; MAP: mean arterial pressure; CMRO _2_: cerebral metabolic rate of oxygen; CMR: the cerebral metabolic rate; *P*aO _2_: O _2_ partial pressure in arterial blood; *P*aCO _2_: CO _2_ partial pressure in arterial blood; H+: hydrogen ion; pH: potential of hydrogen.

The CA can be assessed by measuring CBF response to changes in cerebral perfusion pressure (CPP), ^6^^–^^8^ which is a difference between MAP and mean intracranial pressure (ICP, or MAP-ICP or central venous pressure, CVP, whichever the highest). ^9^ However, CBF can also be influenced by combinations of O _2_ and carbon dioxide (CO _2_) tensions in the blood ( Figure 2). ^10^^,^^11^ CBF variations are measured after a ‘steady state’ change in MAP (such as before and after starting a vasopressor infusion) and dynamic CA, which is defined as a response to rapid changes in MAP (such as deflation of a blood pressure cuff). ^12^ Rather than measuring two distinct physiological mechanisms, ^13^ (static and dynamic), the CA expresses the relationship between MAP and CBF velocity over different timeframes. ^14^ In this review, using CA and CBF preservation, we reappraise the risks and benefits of therapeutic O _2_ administration in patients with brain ischemia and other clinical situations that require supplemental O _2_ as part of routine clinical care.

## Methods

We searched PubMed for English language abstracts, using searching terms such as “oxygen therapy” AND “cerebral autoregulation” OR “ischemic preconditioning” OR “anesthetic neurotoxicity” OR “ischemic stroke” OR “traumatic brain injury” OR “anesthesia”, from 1975 to October 2021. We chose open and blinded studies, reviews and meta-analyses, and available commentaries and editorials related to MESH terms. Because of the nature of the review (narrative, clinical-experience oriented, translational research motivated), an international criteria/PRISMA is not included, and studies are not graded formally by the level of evidence.

## Oxygen: its history, salubrious properties, other dangerous stories

### A brief history of oxygen

Carl Wilhelm Scheele was the first scientist to discover O _2_ in 1771 by heating mercuric oxide, silver carbonate, and magnesium nitrate, but he did not publish his finding till 1777. Meanwhile, in 1774, Joseph Priestley reported that O _2_ allowed a candle to burn more brightly and has been given credit for O _2_ discovery. ^15^ The application of O _2_ in medicine was first reported by Antoine Lavoisier, who described the role of O _2_ in human respiration. In 1798, the Pneumatic Institute (Bristol, England), started O _2_ distribution for treating patients with asthma and congestive heart failure. ^15^ In 1880 with the development of O _2_ cylinders for storage and transport, and in the 1900s with the invention of nasal cannulas and masks, O _2_ therapy for respiratory pathology became a routine clinical practice. ^16^^–^^18^

The application of hyperbaric O _2_ utilization is one of the most advanced applications of O _2_ therapy; it began in 1662 before O _2_ discovery when a British physician Henshaw compressed air in a closed chamber. ^19^ In the late 1800s, specially designed hyperbaric O _2_ chambers were built (the first one in North America around 1860). ^19^ In 1972, Takuo Aoyagi invented pulse oximetry, allowing clinicians to measure continuous peripheral O _2_ saturation and better guide O _2_ therapy in a clinical context. ^20^

### Physiological role of oxygen

Under physiological conditions, 98% of inspired O _2_ is transported in the blood bound to hemoglobin, while the remaining 2% is freely dissolved in the plasma. The bonded O _2_ to hemoglobin known as oxyhemoglobin increases the partial pressure ( *P*O _2_) and oxyhemoglobin saturation in the blood. ^21^ Oxygen in the blood comes from lung inhaled air which is transported to organs and cells utilized for energy production essential for organ function. ^22^ Nutrients such as carbohydrates, proteins, and fats are initially broken down into substrates which entered the tricarboxylic acid (TCA) cycle ^23^ and are converted into nicotinamide adenine dinucleotide (NADH) and flavin adenine dinucleotide (FADH2). The NADH and FADH2 entered the electron transport chain (ETC), which is composed of several protein complexes located in the inner membrane of the mitochondria. Both NADH and FADH2 donate electrons to the ETC, which carries them down while allowing protons to be pumped into the inner membrane space. ^23^ After electrons reach complex IV, the O _2_ accepts the electrons and is reduced to water. ^24^

The protons pumped in the intermembrane space create a mitochondrial membrane potential to convert adenosine diphosphate (ADP) to adenosine triphosphate (ATP) via a synthase enzyme, the main energy molecule in the body. ^23^ Without O _2_, the ETC would not have a final electron acceptor and the production of ATP would become much less efficient (when the level of oxygen in mitochondria falls to less than 1–2 mmHg, known as Pasteur point, anaerobic metabolism of glucose results in synthesis of lactic acid).

### Indications for oxygen therapy

Oxygen is usually administered under hypoxemia which occurred as a result of a decrease in arterial O _2_ tension. ^25^ Oxygen therapy is beneficial in pathologies that increased O _2_ utilization and/or decreased O _2_ delivery to tissues. ^24^ In patients with acute respiratory failure, supplemental O _2_ remains an essential treatment component, which has significantly improved with advanced O _2_ delivery systems. ^26^

### Use of oxygen in anesthesia

General anesthesia (GA) is a typical clinical situation when O _2_ is commonly administered. GA can indeed decrease O _2_ partial pressure in arterial blood ( *P*aO _2_) *via* multiple mechanisms: functional residual capacity (FRC) decreases after induction of anesthesia due to the diaphragm being cranially displaced in supine position; the decrease in FRC reduces lung compliance and increases airway closure at end-expiration, predisposing to atelectasis and hypoxemia ^27^; moreover, apnea periods during airway manoeuvres can lead to rapid arterial hemoglobin desaturation and hypoxia; therefore, anesthesiologists commonly administer a high inspired O _2_ fraction ( *F*iO _2_) before tracheal intubation and extubation. ^23^^,^^24^^,^^28^

### Adverse effects of oxygen therapy

Despite some benefits that O _2_ supplementation could serve in a variety of clinical scenarios (simple hypoxia, hypoventilation, organ ischemia events, increased O _2_ demand, toxic hypoxia, to cite few), an indiscriminate administration of therapeutic O _2_ at high concentrations and/or for a prolonged time may lead to adverse effects: of those, formation of reactive O _2_ species (ROS) and increased systemic vascular resistance ^29^ could further play a role in generating or contributing for instance to brain injury or other organ injuries. Even a physiological vascular territory where possibly O _2_ may exert by common sense a salubrious effect, like the pulmonary, may indeed negatively affect Hypoxia-induced Pulmonary Vasoconstriction HiPV, therefore causing ventilation/perfusion ( *V*/ *Q*) mismatching. Overall what we have described as the “oxygen paradox”, is also “The Tale of Two Cities”. ^30^

*a. Reactive oxygen species (ROS)*

Physiologically, when O _2_ is utilized in the mitochondrial ETC, only a small amount of ROS is formed by the partial reduction of O _2_. ^31^ Although ROS such as superoxide anion, hydrogen peroxide, and hydroxyl radical can lead to cell damage, their action is usually counteracted by various intra- and extracellular antioxidants such as superoxide dismutase, glutathione, cytochrome c oxidase. ^32^

With an increase in O _2_ supplementation, ROS production is also increased *via* outstrips the available antioxidant levels. ROS causes damage to nucleic acids, proteins, and lipids leading to cell damage and ultimately cell death. ^31^ An excess of O _2_ can also lead to inflammation and possible lung damage. ^29^ In lungs, hyperoxia and ROS stimulate nuclear factor kappa B, which releases plasminogen activator inhibitor-1 and tissue factor. The release of these factors causes activation of the coagulation cascade, which may induce further cell damage. ^21^ Importantly, ROS have been shown to play a role in both neuronal death and neurovascular recovery after cerebral ischemia. ROS, interleukin-1β and hydrogen peroxide (H _2_O _2_) activate p38-mitogen-activated protein kinase, which is associated with protein oxidation and damage in post-ischemic rodent brains. ^33^ Intriguingly, while ROS mediates neuronal damage during the early phase of ischemia, in later phases it mediates vascular endothelial growth factor (VEGF) synthesis, angiogenesis, and recovery in the post-ischemic brain. ^34^

*b. Oxygen-mediated vasoconstriction and other vaso-mediated mechanisms*

ROS production linked to excess O _2_ inhibits cyclooxygenase, decreasing prostaglandins, and thereby lead to vasodilation. ^35^ The ROS superoxide anion inactivates nitrous oxide (NO), a vasodilator, through various mechanisms. ^36^^,^^37^ A mechanism by which hyperoxia causes vasoconstriction is by converting arachidonic acid to 20-hydroxy-eicosatetraenoic acid (20-HETE). ^38^ These mechanisms have been described causing systemic vasoconstriction and decreased perfusion to almost all organs, ^36^ supposedly with the exception of the lungs and the placenta: yet HIPV may be disrupted by O _2_ and similar concerns have been raised for adequate placental flow and the autoregulation of optimal fetal perfusion. ^39^ Hyperoxia-mediated vasoconstriction may affect more the microvasculature rather than larger vessels: for example, the large coronary arteries do not constrict under hyperoxic conditions ^40^; also, a decrease in prostaglandin levels (affected by ROS production) normally cause vasodilation mainly in the microvasculature. ^41^

*c. Oxygen therapy post-cardiac arrest and myocardial infarction*

Supplemental O _2_ administration after myocardial infarction (MI) was originally adopted to attenuate tissue ischemia. However, it has been postulated that ROS can in fact cause tissue injury and excess O _2_ can lead to decreased peripheral coronary perfusion, which in turn may adversely affect patient outcomes. ^40^ A Cochrane review and meta-analysis showed that patients with ST-elevation myocardial infarction (STEMI) or non-STEMI (NSTEMI) treated one hour of inhaled O _2_ within 24 hours of symptom onset, exhibited an increase in infarct size [measured as increased creatine kinase (CK)] as compared to patients treated with air. ^42^ However, a randomized control trial was unable to show that O _2_ therapy after MI caused any difference in mortality. ^43^

The effect of O _2_ therapy on post-ventricular fibrillation arrest and subsequent return of spontaneous circulation (ROSC) has also been reported. ^44^ In 28 patients, randomized to receive either 0.3 *F*iO _2_ or 1 *F*iO _2_, their serum samples collected at 24 and 48 hours were analyzed for neuron specific enolase (NSE) and protein S-100, showed signs of neuronal injury. Although statistically there was no difference in the NSE and protein S-100 levels between the two groups, a higher NSE levels were reported in patients who received 1 *F*iO _2_ and did not undergo therapeutic hypothermia. ^44^ There is some evidence suggesting that O _2_ may not improve outcomes after MI and even worsen the outcome, increasing the risk of neurological injury after cardiac arrest and return of spontaneous circulation (ROSC).

*d. Oxygen therapy after ischemic stroke*

The role of hyperoxia in patients with a previous stroke has not been entirely and clearly elucidated. In acute ischemic stroke, the occluded blood vessels disrupt brain O _2_ delivery causing hypoxia/ischemia and a main infarction area, as well as another area called penumbra, surrounded by healthier tissues amenable of being rescued if cerebral perfusion and oxygenation are restored in a timely fashion. Besides the direct vascular mechanisms of vascular patency though, there are many others which have been discussed so far: those include ROS production and nutrients supply, as well as mechanisms related to the vascular integrity per se or “barriers” integrity, i.e. the blood brain barrier being one of them and discussed later in the paragraph Changes in the Neurovascular Unit after Ischemic Stroke.

Animal studies reported that an early administration of supplemental O _2_ after stroke may improve clinical outcomes ^10^^,^^45^: however, hyperoxia may physiologically induce cerebral vasoconstriction, which is the other main topic of our narrative review. ^46^ In a similar clinical scenario, the role of O _2_ and neuronal survival/remodeling has been questioned in other clinical scenarios such as neonatal asphyxia. ^47^

## Cerebral autoregulation

### The neurovascular unit

The neurovascular unit (NVU) includes multiple cell types (neurons, interneurons, astrocytes, microglia, smooth muscle cells, pericytes, endothelial cells) and extracellular matrix which are closely associated with cerebral vasculature and their interaction with each other to maintain the blood-brain barrier (BBB) and regulate CBF. ^48^^,^^49^ A tight junction between endothelial cells of the blood vessels and BBB’s selective permeability control *via* NVU pericytes proteins is crucial. The NVU is also involved in vascular tone regulation and an increased in neuronal activity leading to increased CBF and thereby increased oxygenation. ^50^

### Mechanisms of cerebral autoregulation

Cerebral autoregulation is also involved in vasoconstriction and vasodilation of cerebral arterioles *via* myogenic, neurogenic, and metabolic regulation ( Figure 2). ^51^^,^^52^ Myogenic regulation acts *via* smooth muscle cell contraction in the wall of blood vessels, ^53^ is the key to maintaining CBF when MAP is outside the autoregulation range, whereas within the autoregulation range neurogenic regulation by the NVU plays a major role. ^52^ As part of the NVU, neurons respond to decreased glucose and hypoxia by generating molecular signals, such as glutamate, to communicate with interneurons and astrocytes. ^54^ Astrocytes on their part can alter vascular tone via prostaglandins, ATP, NO, and lactate. ^48^^,^^54^

### Roles of O _2_ and CO _2_ in autoregulation

Cerebral blood flow is primarily affected by blood gas concentrations, including O _2_, CO _2_, and nitric oxide (NO). ^55^^–^^57^ Hypoventilation causes an increase in arterial CO _2_ tension (hypercapnia) and leads to vasodilation resulting an increase in CBF, whereas inhalation of 100% O _2_ reduces CBF by 10–15%. ^58^^,^^59^ In essence, CBF is directly influenced by blood oxygenation levels and vice versa. Under hypoxic conditions (or hypercapnia), autoregulation initiates activation of mechanisms leading to vasodilation, whereas hyperoxia (or hypocapnia) increases O _2_ bioavailability as CBF decreases due to vasoconstriction ( Figure 2). ^10^^,^^11^

The partial pressure of O _2_ ( *P*aO _2_) and partial pressure of carbon dioxide ( *P*aCO _2_) can also have a combined effect, depending on their respective partial pressures. Ogoh *et al*. studied the effect of O _2_ therapy on dynamic CA by exposing nine healthy volunteers to four respiratory interventions: normoxia (0.21 *F*iO _2_), isocapnic hyperoxia (0.4 *F*iO _2_), isocapnic hypoxia (0.14 *F*iO _2_), and hypocapnic hypoxia (0.14 *F*iO _2_). ^60^ He used transcranial Doppler to measure middle cerebral artery blood velocity to determine CA. The CA was impaired with normocapnic hypoxia; however, it improved with mild hypocapnic hypoxia. He concluded that hypocapnia-caused vasoconstriction led to improved CA, thus outweighing the negative effect of hypoxia on CA. ^60^

### Changes in cerebral circulation after ischemic stroke

In the event of CPP reduction such as an acute ischemic stroke, a compensatory mechanism starts to preserve O _2_ and nutrient supply to the brain. Based on a human study (MRI was used to assess O _2_ extraction fraction (OEF) in patients with various degrees of middle cerebral artery (MCA) stenosis or acute stroke), ^61^ it was found that the increase in OEF from baseline was higher in the severe MCA stenosis group compared to the mild stenosis group, where collateral circulation allows for some perfusion distal to the lesion. Patients with severe MCA stenosis have a greater reduction in CBF and little collateral circulation, necessitating other mechanisms such as improving OEF to increase brain oxygenation. ^61^

In animal studies, occluding the MCA caused an increase in CBF and OEF one hour after occlusion; however, at 2–3 hours post-occlusion CBF and OEF had both decreased. ^62^ These findings suggest that soon after MCA occlusion compensatory mechanisms attempt to maintain cerebral perfusion and oxygenation. However, at later stages of MCA occlusion these compensatory mechanisms, and presumably cerebral oxygenation, decrease. ^62^

To summarize, to maintain O _2_ and nutrient delivery to the brain during acute stroke, CA causes arterioles to dilate, therefore increasing CBF. Once this compensatory mechanism has been used up, O _2_ extraction fraction (OEF) can increase significantly to keep O _2_ metabolism running, but at maximal OEF, continuing or deteriorating CBF reduction may eventually lead to cell death. ^62^

### Changes in the neurovascular unit after ischemic stroke

Ischemic stroke and subsequent cerebral hypoxia can cause disruption of the NVU and damage to the BBB *via* the hypoxia-inducible factor 1 (HIF1) transcription factor, that is constituted of two subunits, the alpha being sensibly regulated by oxygen. ^63^ Additionally, HIF1-alpha and cytokines such as TNF-alpha and IL-1B lead to the activation of matrix metalloproteinases (MMPs) which break down the BBB and increase its permeability. ^64^ A decrease in ATP production and failure of enzymes to maintain normal ion gradients cause endothelial cell swelling and BBB dysfunction. ^65^ Ischemic stroke leads to degradation of extracellular proteins, detachment of pericyte, astrocyte, and microglia activation. ^49^ These changes in the BBB and the NVU allow for peripheral immune cells to enter the brain causing inflammatory stress. ^66^ Loss of BBB integrity also leads to vasogenic edema and increases the risk of hemorrhagic transformation, which can worsen brain damage after stroke. ^67^

## Anesthesia and autoregulation

General anesthesia (GA) can directly impact cerebral autoregulation, therefore potentially inducing a direct organ damage, in particular brain damage. Besides that, GA could also be cause of neurotoxicity due cell-toxicity, yet anesthesia may also be responsible for molecular mechanisms of neuroprotection therefore modulating neurotoxicity: these mechanisms are encompassed for instance in the same processes responsible for cell and organ protection after ischemic precondition and postconditioning.

A further consideration pertains the use of O _2_ not simply for the preparation prior to general anesthesia in otherwise healthy subjects, yet in patients during or after cardiac arrest or ischemic stroke that require therapeutic use of O _2_, and in particular when they require procedures involving GA: at a time when CBF and oxygenation are compromised, GA may pose another threat to organs health or possibly be a reason of organ protection. Here we briefly review the available evidence around some of the potential effects of GA on brain tissue and CA.

### Anesthesia-induced neurotoxicity

Volatile anesthetics can cause neurotoxicity, especially in neonatal rats and non-human primates, although the exact mechanism remains under investigation. They can lead to neuroapoptosis, neurodegeneration, and long-term neurocognitive deficits in animal models through N-methyl-D-aspartate (NMDA) receptor antagonism and GABA receptor activation. ^68^ Ultimately, they can also create oxidative stress in the mitochondria, leading to ROS production which triggers a chain of events causing apoptosis, ^69^ and contribute to neurotoxicity. ^69^^–^^71^

Both *in vitro* and *in vivo* studies have shown evidence of propofol-induced cell death. ^72^^,^^73^ The mechanism of propofol-induced neurotoxicity is thought to be mediated by multiple pathways including apoptosis, decreased neurogenesis, disruption of dendrite formation, neuroinflammation, Ca ^2+^ signalling, microRNAs, and activation of p75 neurotrophic receptor. ^72^^,^^73^

### Anesthesia-induced neuroprotection

Volatile anesthetics also, seem to play a role in organ protection through ischemic preconditioning and postconditioning, shown by cardiac animal studies a similar mechanism has been found in brain cells. ^74^ Sevoflurane is involved in neuroprotection when administered at specific times before ischemia–reperfusion. Sevoflurane-induced energy preservation decreases both focal and global ischemia and improves outcomes. ^75^ Additionally, sevoflurane and other volatiles inhibit glutamate receptors which are normally stimulated to cause cell injury during ischemia. ^75^

Propofol also induces neuroprotection through ischemic preconditioning and postconditioning via a different mechanism from volatile anesthetics. ^75^ Propofol regulates cytochrome c, Cx43, UCP2, and mitochondrial DNA (mtDNA) transcription, which all play a role in neuroprotection after ischemia. It protects the integrity of the mitochondrial membrane during ischemia, preventing cytochrome c detachment and activation of the apoptosis pathway, thereby preventing neuronal cell death. ^75^

### Effect of anesthesia on cerebral autoregulation

While the hemodynamic effects of commonly used anesthetics such as sevoflurane, propofol, and dexmedetomidine are well known, their effect on CA is less extensively studied. Usually the administration of sevoflurane at 1 minimal alveolar concentration (MAC), while it somewhat decreases MAP, overall it maintains CBF: CA is overall preserved as shown by Juhász *et al*. in 29 patients cohort who underwent GA. ^76^ Using dexmedetomidine with sevoflurane and nitrous oxide anesthesia would also appear to not affect CA. ^77^ McPherson *et al*. reported fentanyl has no known impact on CA. ^78^

General anesthesia using propofol with target-controlled infusion also maintains CA and CO _2_ reactivity through a balance between vasodilation and vasoconstriction. ^73^^,^^79^ CA was unchanged in patients undergoing GA with propofol-remifentanil infusion but decreased in those receiving high-dose sevoflurane. Additionally, higher CO _2_ levels did not affect CA in the propofol-remifentanil group, while the high-dose sevoflurane group experienced a further reduction in CA. ^80^ These studies suggest that CA is maintained in patients receiving propofol at doses required for GA.

## Cerebral autoregulation and imaging: a window in the pathophysiology of post-stroke disruption

Currently, CA can be assessed by raising BP by ~10 mmHg *via* a pharmaceutical agent while measuring intracranial pressure (ICP), or by continuous monitoring of brain tissue *P*O _2_ using an intraparenchymal probe: a decrease, or no change, in ICP indicates an intact CA, while an increase in ICP suggests compromised CA. ^81^ Non-invasive techniques such as transcranial Doppler (TCD) and near-infrared spectroscopy (NIRS) can be used to assess cerebral reactivity, autoregulation, and neurovascular coupling; however, TCD failed to provide more global CBF, whereas NIRS is limited by the infrared penetration power in the deepest parts of the brain. Both methods have accuracy, sensitivity, and reproducibility issues, and lack a complete cerebral hemisphere coverage. ^82^^,^^83^ Other methods, such as positron emission tomography (PET), single-photon emission computed tomography (SPECT), and computed tomography (CT) can compute CBF, cerebral blood volume (CBV), mean transit time (MTT), and O _2_ extraction fraction (OEF); however, they require a radioactive tracer with a short half-life. ^84^^–^^86^ Gas-challenge magnetic resonance imaging (MRI) is emerging as a viable option to probe CA by measuring the change in CBF, the cerebral metabolic rate of oxygen (CMRO _2_), and OEF. ^87^^–^^89^

Inhalation of pure O _2_ or carbogen (a mixture of 3–5% CO _2_ and 95–97% O _2_) changes blood oxygenation in capillaries and veins by altering oxyhemoglobin *versus* deoxyhemoglobin content. This manifests as a change in T2*-weighted MRI signal, which is the principal blood oxygenation level-dependent (BOLD) imaging contrast (see Figure 1, panel B as a hypothetical model of O _2_ inhalational effect assessed by MRI). ^90^^,^^91^

Gas challenge images have been used to compute vessel size. ^92^ Under physiological conditions, the arterial blood is 98% oxygenated resulting in a small difference in magnetic susceptibility between arteries and tissue. With O _2_ inhalation, an increase in *F*iO _2_ from 0.21 to 1.0 leads to a significant change in BOLD contrast. In ischemic stroke patients, Donahue *et al*. applied carbogen challenge MRI to evaluate cerebrovascular reserve capacity in patients with intracranial stenosis. ^93^ Carbogen increases the fractional concentration of inspired CO _2_ ( *F*iCO _2_) and NO-mediated vasodilation, which in turn increase MRI signal intensity. In contrast to O _2_, carbogen challenge MRI has been extensively applied to study several pathologies such as malignancy, hypoxia, Alzheimer’s, retinopathy, type 2 diabetes, hepatic fibrosis, and chronic kidney disease. ^94^^–^^97^

Gas-challenging MRI is emerging as a non-invasive mainstream method to study neurophysiology. In a healthy brain, a hypercapnia-induced increase in CBF affects the CMRO _2_ and OEF. Several studies reported both CMRO _2_ and OEF are significantly altered in patients with stroke, tumor, and cerebrovascular degenerative disorder. ^98^^,^^99^ Both hypercapnia and hyperoxia induce CBF changes leading to BOLD MRI signal by affecting venous blood deoxyhemoglobin concentration has also been applied to compute these two critcal functions. ^100^^–^^102^

A localized or global change in oxy- and deoxyhemoglobin concentrations in the brain can alter magnetic susceptibility and blood transverse relaxation time (T2 and T2* relaxation time); this has been recently applied to compute OEF. A change in blood gases can alter magnetic susceptibilities: these changes can be measured by quantitative mapping (QSM), an advanced MRI signal processing method that uses both the magnitude and phase component of a gradient echo MRI signal.

A magnetic susceptibility difference between the veins and the surrounding tissue has been applied to estimate venous O _2_ saturation. Uwano *et al.* and Zaitsu *et al*. applied QSM to measure OEF and validated it with PET imaging, a gold standard to measure OEF. ^103^^,^^104^ Previously, Zhang *et al*. used caffeine and hyperventilation as stimuli to alter susceptibility via a change in oxy- *versus* deoxyhemoglobin ratio and measured OEF and CMRO _2_ using QSM. ^105^^,^^106^ However, because both caffeine and hyperventilation induce vasoconstriction, the CBF reduction results in a smaller magnetic susceptibility difference between the oxy- *versus* deoxyhemoglobin creating weaker MRI contrast. ^105^^,^^106^ To obviate those limitations, Ma *et al*. recently applied a carbogen inhalation vasodilator challenge: it increased CBF and resulted in a larger magnetic susceptibility difference between the oxy- *versus* deoxyhemoglobin, creating stronger MRI contrast. ^107^

To summarize, CA can be assessed by various methods, all of which have specific limitations. Emerging non-invasive imaging techniques relying on the gas challenge look promising for the study of cerebral pathophysiology.

## Conclusions

While exogenous O _2_ administration is still part of routine clinical management and therapeutic algorithms, it remains unclear at present whether this practice should continue indiscriminately, considering its potential toxic effects. Specifically, despite the clear dependence of the human brain on a constant O _2_ and nutrient supply, a compelling clinical and experimental body of evidence questions the utility of excess O _2_ in acute and post-recovery brain ischemia. Many of these findings have been the object of translational research, particularly in the field of neurophysiology imaging. It may be prudent and possibly best practice to limit exogenous O _2_ administration in the clinical setting to the shortest period required, and consider reducing inspired O _2_ fractions in patients more predisposed to CA disruption, such as post-stroke recovered patients.

## Authors’ contributions

PG: designed the work, contributed to literature search, collected data, interpreted data and wrote the manuscript

NF: designed the work, contributed to literature search, collected data, interpreted data, wrote the manuscript

DS: designed the work, literature search, collected data, interpreted data, edited the manuscript and figures

MH: contributed to literature search, wrote the manuscript, interpreted data, edited the manuscript and figures

DC: initiated this report, designed the work, collected data, interpreted data, edited the manuscript and figures

All authors read and approved the final manuscript.

## Data Availability

All data underlying the results are available as part of the article and no additional source data are required.
