# Supplementary material for: Tale of Two Cities: narrative review of oxygen
Source: F1000Res. 2023 May 9;12:246. Originally published 2023 Mar 6. [Version 2] doi: 10.12688/f1000research.130592.2 (PMC10189297; doi:10.12688/f1000research.130592.2)
Supplement: Peer review history

# Peer review: Reviewer response for version 2

**DOI:** 10.5256/f1000research.147777.r172555

**Published:** 2023-05-16

**Authors:** Carlo Pancaro

**Affiliations:** 1 Department of Anesthesiology, University of Michigan Health System, Ann Arbor, Michigan, USA

I recommend to approve this version since the authors addressed all issues.

Is the review written in accessible language?

Yes

Are all factual statements correct and adequately supported by citations?

Yes

Are the conclusions drawn appropriate in the context of the current research literature?

Yes

Is the topic of the review discussed comprehensively in the context of the current literature?

Yes

Reviewer Expertise:

Neuroscience, Obstetric Anesthesiology, Cardiovascular Physiology

I confirm that I have read this submission and believe that I have an appropriate level of expertise to confirm that it is of an acceptable scientific standard.

# Peer review: Reviewer response for version 1

**DOI:** 10.5256/f1000research.143362.r165648

**Published:** 2023-05-02

**Authors:** Ivana Budic

**Affiliations:** 1 Faculty of Medicine, University of Niš, Niš, Serbia

The manuscript submitted by Gullapalli *et al.* is a narrative review composed by a team of anesthesiologists, intensivists and neurobiologist, intended for broad audience. It is not an experts review per se, yet the authors organized the different paragraphs accessing several key reference works based on their own experience. A very interesting and novel perspective, is the graphical content, which adds an original aspect to the original approach. The authors started the review emphasizing a general cover on the topic and they search from PubMed of English language abstracts, using adequate searching terms, from 1975 to October 2021.

 They extracted the essence of several good articles about the use of oxygen in various pathophysiological conditions as well as during anaesthesia, emphasizing the contradictory effects of its application, which justifies the title of the paper "Tale of Two Cities". The use of oxygen has been questioned indeed on the beneficial effects in different clinical scenarios as well as in controlled preclinical studies. The authors, even if briefly, address a clinical novel evaluation of the long-term vascular reactivity and cerebral autoregulation after stroke, while exposing patients to administration of oxygen.

 Is the narrative review too general, too long, or even dispersive? On a general knowledge standpoint and trying to offer a scholastic resource for non-experts it is not; it still offers some expert view points on critical topics, like neuroapoptosis and cellular mechanisms of injury as well as neuroimaging and coupling. It does build a case for what it is known and moving in a hypothetical model testing the safety of clinical practice.

 I had the chance to read Dr Pancaro notes about the manuscript and his peer-review of the paper is in line with my own assessment: I agree with the assessment that it is an interesting reading with only few minor changes to be completed. I congratulate the authors for a simple yet unique presentation, and the nice artworks.

Is the review written in accessible language?

Yes

Are all factual statements correct and adequately supported by citations?

Yes

Are the conclusions drawn appropriate in the context of the current research literature?

Yes

Is the topic of the review discussed comprehensively in the context of the current literature?

Yes

Reviewer Expertise:

Preventive, therapeutical and ethical approach to the preclinical and clinical research of genes and modulators of redox cell signalization in immune, inflammatory and proliferatory cell response

I confirm that I have read this submission and believe that I have an appropriate level of expertise to confirm that it is of an acceptable scientific standard.

# Response

**Published:** 2023-05-02

**Authors:** Davide Cattano

**Affiliations:** McGovern Medical School, UTHealth, USA

We are grateful to the reviewer for the kind comments and will proceed on changes as recommended.

# Peer review: Reviewer response for version 1

**DOI:** 10.5256/f1000research.143362.r165646

**Published:** 2023-04-11

**Authors:** Carlo Pancaro

**Affiliations:** 1 Department of Anesthesiology, University of Michigan Health System, Ann Arbor, Michigan, USA

The authors should be commended for the nice, well written review. Here are a few small suggestions:

1. Page 3 – I would consider to adopt the Definition of CPP = **MAP** – **ICP** or **CVP** (whichever is highest).
2. Page 5 - Without O _2_, the ETC would not have a final electron acceptor and the production of ATP would become much less efficient - Here I would briefly mentioned the Pasteur point where the cell metabolism switches from aerobic to anaerobic leading to lower ATP production. Oxidative metabolism of each glucose molecule results in production of 38 ATP. When the level of oxygen in mitochondria falls to around 1–2 mm Hg (Pasteur point), anaerobic metabolism of glucose results in synthesis of lactic acid with a consequent generation of 2 ATP per glucose only.
3. Page 5 – Change HIPV to (HPIV).
4. Page 5 - Intriguingly, while ROS mediates neuronal damage during the early phase of ischemia, in later phases it mediates vascular endothelial growth factor –You might want to add “synthesis” after endothelial growth factor.
5. Beginning of Page 6 – Change “and thereby lead to” to “and thereby leads to” or “and thereby leading to”.
6. Page 6 – Change “In a 28 patients” to “In 28 patients”.
7. Page 6 – FiO _2_ 1.0 - Since in this case 1 represents a fraction and there is no number greater than 1, I would eliminate the ".0" – I would also do the same for the rest of the review.
8. Page 6 – change “did not underwent” to “did not undergo”.
9. Page 6 - (neurons, interneurons, astrocytes, smooth muscle cells, pericytes, endothelial cells) – I would add Microglia to this list.
10. Page 7- HIF1a – I would spell the alpha here and write HIF1-alpha.

Is the review written in accessible language?

Yes

Are all factual statements correct and adequately supported by citations?

Yes

Are the conclusions drawn appropriate in the context of the current research literature?

Yes

Is the topic of the review discussed comprehensively in the context of the current literature?

Yes

Reviewer Expertise:

Neuroscience, Obstetric Anesthesiology, Cardiovascular Physiology

I confirm that I have read this submission and believe that I have an appropriate level of expertise to confirm that it is of an acceptable scientific standard, however I have significant reservations, as outlined above.

# Response

**Published:** 2023-04-11

**Authors:** Davide Cattano

**Affiliations:** McGovern Medical School, UTHealth, USA

Thank you Dr Pancaro for the valuable comments and suggested revisions. They will improve the reading and understanding of outlined information.
